# Ezetimibe and Simvastatin Reduce Cholesterol Levels in Zebrafish Larvae Fed a High-Cholesterol Diet

**DOI:** 10.1155/2012/564705

**Published:** 2012-05-30

**Authors:** Ji Sun Baek, Longhou Fang, Andrew C. Li, Yury I. Miller

**Affiliations:** Department of Medicine, University of California, San Diego, La Jolla, CA 92093, USA

## Abstract

Cholesterol-fed zebrafish is an emerging animal model to study metabolic, oxidative, and inflammatory vascular processes relevant to pathogenesis of human atherosclerosis. Zebrafish fed a high-cholesterol diet (HCD) develop hypercholesterolemia and are characterized by profound lipoprotein oxidation and vascular lipid accumulation. Using optically translucent zebrafish larvae has the advantage of monitoring vascular pathology and assessing the efficacy of drug candidates in live animals. Thus, we investigated whether simvastatin and ezetimibe, the principal drugs used in management of hypercholesterolemia in humans, would also reduce cholesterol levels in HCD-fed zebrafish larvae. We found that ezetimibe was well tolerated by zebrafish and effectively reduced cholesterol levels in HCD-fed larvae. In contrast, simvastatin added to water was poorly tolerated by zebrafish larvae and, when added to food, had little effect on cholesterol levels in HCD-fed larvae. Combination of low doses of ezetimibe and simvastatin had an additive effect in reducing cholesterol levels in zebrafish. These results suggest that ezetimibe exerts in zebrafish a therapeutic effect similar to that in humans and that the hypercholesterolemic zebrafish can be used as a low-cost and informative model for testing new drug candidates and for investigating mechanisms of action for existing drugs targeting dyslipidemia.

## 1. Introduction

 In 2009, we suggested, for the first time, to use zebrafish as a model organism to study specific vascular events relevant to development of human atherosclerosis [1]. Feeding adult zebrafish a high-cholesterol diet (HCD) results in hypercholesterolemia, with total plasma cholesterol reaching 800 mg/dL, profound lipoprotein oxidation, and formation of vascular lesions, resembling human fatty streaks. HCD-fed adult zebrafish have been used by Cho and coworkers to study effects of food supplements and sweeteners on plasma cholesterol levels and CETP activity [2–4]. The optical transparency of zebrafish larvae within first 30 days post fertilization (dpf) enables microscopic monitoring in live animals of vascular lipid accumulation, recruitment of myeloid cells, formation of macrophage foam cells, disorganization of the endothelial cell layer, and increases in vascular PLA_2_ activity and vascular permeability—all induced by a short (5–14 days) HCD feeding [1, 5]. Furthermore, our recent report demonstrates applications of a transgenic *hsp70:IK17-EGFP* zebrafish, with conditional expression of the EGFP-conjugated antibody IK17 specific to oxidized LDL, in studying *in vivo* lipoprotein oxidation and in testing therapeutic effects of antioxidants [5].

 Because feeding zebrafish a HCD leads to extensive lipoprotein oxidation, we further characterized the oxidized lipid milieu in HCD-fed larvae [6]. A 2-week HCD feeding resulted in up to 70-fold increases in the levels of oxidized cholesterol esters, oxidized phospholipids, and lysophospholipids in zebrafish homogenates. Remarkably, specific oxidized lipid molecules detected in HCD-fed zebrafish larvae using liquid chromatography-mass spectrometry were identical to those found in human and mouse atherosclerotic lesions [6]. Lipoproteins isolated from HCD-fed larvae activated mouse macrophages *in vitro*, with the pattern of ERK1/2, JNK, and Akt phosphorylation and cell spreading similar to that induced by minimally oxidized human LDL [6].

Thus, our work and the work of others suggest that hypercholesterolemia and lipoprotein oxidation achieved in zebrafish and characteristics of vascular lipid accumulation and inflammatory processes, relevant to human atherogenesis, make the zebrafish model an attractive *in vivo* system for testing novel therapeutic approaches and studying the mechanisms of existing drugs. The goal of the present study was to investigate whether simvastatin and ezetimibe, the principal drugs used for management of hypercholesterolemia in humans, would also reduce cholesterol levels in HCD-fed zebrafish larvae.

## 2. Materials and Methods

### 2.1. Zebrafish Maintenance and Feeding

 Wild-type (AB) zebrafish embryos were obtained by *in vitro* fertilization and natural spawning of adults maintained at 28°C on a 14/10 hour light/dark cycle and staged as described [7]. Zebrafish larvae were fed twice a day, starting at the 5th day post fertilization (dpf), with either control diet (Golden Pearls, 100–200 mm size from Brine Shrimp Direct) or HCD (4% cholesterol dissolved in diethyl ether added to Golden Pearls) for 14 days, as described in our previous work [1]. All animal studies were approved by the Animal Care and Use Committee of the University of California, San Diego.

### 2.2. Treatment with Ezetimibe and Simvastatin

 Ezetimibe (Santa Cruz Biotechnology, cat. no. sc-205690) was added directly to the fish tank water at concentrations of 0.1–50 *μ*M. This concentration range was chosen based on previous reports that 50 *μ*M ezetimibe prevented approximately 75% of intestinal cholesterol absorption in zebrafish [8] and our preliminary studies showing a high efficiency of ezetimibe at lower doses. Simvastatin (Cayman Chemicals, cat. no. 10010344) was initially added to the fish tank water, in the same manner as it was done with ezetimibe. We observed that even at low doses of simvastatin, added directly to water, it was toxic to zebrafish larvae. Thus, we changed the protocol and mixed simvastatin into the fish food using the same procedure as described above for making a HCD. Simvastatin was added at 0.1–50 *μ*g per gram food. We estimated that 20 larvae in a fish tank consumed approximately one half of 1.5 mg food administered 2 times a day. Given that larvae at this age weigh 0.8–1.0 mg, we calculated that the dose was between 9 and 3,750 ng simvastatin per mg body weight. This translates into an equivalent of 0.6–260 mg simvastatin per 70 kg body weight given to humans. Since clinical doses of simvastatin range from 5–80 mg, we consider doses of simvastatin administered to zebrafish larvae being roughly within a therapeutic range used to treat patients. The ezetimibe and/or simvastatin treatments described above did not affect zebrafish feeding behavior, swimming pattern, nor their growth (apparent body size).

### 2.3. Lipid Extraction from Zebrafish Homogenates and Cholesterol Measurements

 At the end of the feeding/treatment period, 20 zebrafish larvae in each experimental group were euthanized by exposure to 0.05% tricaine (Sigma). Abdomens containing undigested food were removed, and the remaining bodies were pooled and gently homogenized in 200 *μ*L of ice-cold PBS in an eppendorf tube using a plastic pestle. After spinning down tissue debris, the supernatant representing body fluids was used for further analysis. Protein content in the homogenates was determined using the Bradford assay with a BCA Protein Assay kit (Pierce). Total lipid was extracted from zebrafish homogenates as we previously described [6]. In brief, the tissue homogenates were supplemented with 50 *μ*g stigmasterol, an internal standard to control for recovery of extracted sterols. Total lipid extraction was performed with 1 : 2 methanol/dichloromethane. Cholesterol esters were saponified and total free cholesterol and stigmasterol were measured with a Shimadzu GC-2014 gas chromatograph using a 30 m × 0.25 mm (i.d.) ZB-5HT inferno capillary column, film thickness 0.2 *μ*m (Phenomenex). Cholesterol and stigmasterol standards were analyzed in parallel with experimental samples.

### 2.4. Statistics

 Student's *t*-test was used to analyze differences between means of 2 groups. *P* < 0.05 was used as the significance threshold.

## 3. Results and Discussion

**Figure 1:**
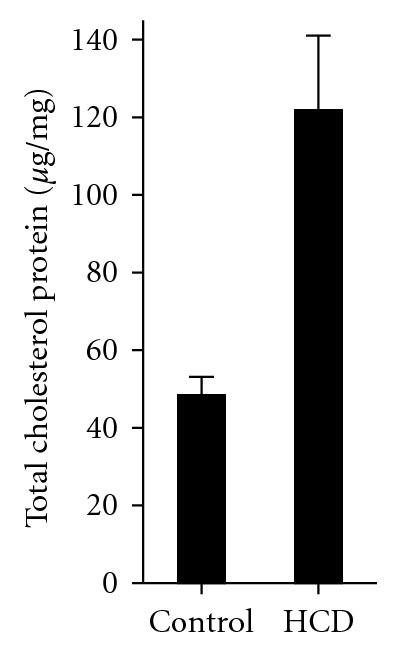
Total cholesterol levels in HCD-fed zebrafish larvae. Zebrafish larvae were fed control or high-cholesterol diets starting at the 5th dpf and continued for 14 days. Total cholesterol levels are expressed in *μ*g cholesterol per mg protein of larvae lysate. Mean ± SEM from 6 independent experiments; 15–20 larvae were pooled for each experimental data point in each individual experiment. *P* < 0.005.

 We have previously reported that plasma cholesterol levels in adult zebrafish rise from 200 to 800 mg/dL following 12 weeks of HCD feeding. Zebrafish larvae are too small to draw blood. Thus, we measured cholesterol levels in body fluids isolated from the larvae from which abdomens were removed and remaining bodies were gently homogenized. The homogenates were centrifuged to pellet tissue debris, and supernatants were used to extract total lipid and measure cholesterol using a gas chromatography method. A 2-week HCD feeding resulted in a 2.5-fold increase in cholesterol levels in zebrafish larvae (Figure 1). These results suggest that the experimental conditions used in experiments with zebrafish larvae reported in our earlier work [1, 5, 6] lead to significant elevations in cholesterol levels in larvae body fluids, comparable with hypercholesterolemia reported for adult zebrafish fed a HCD [1].

**Figure 2:**
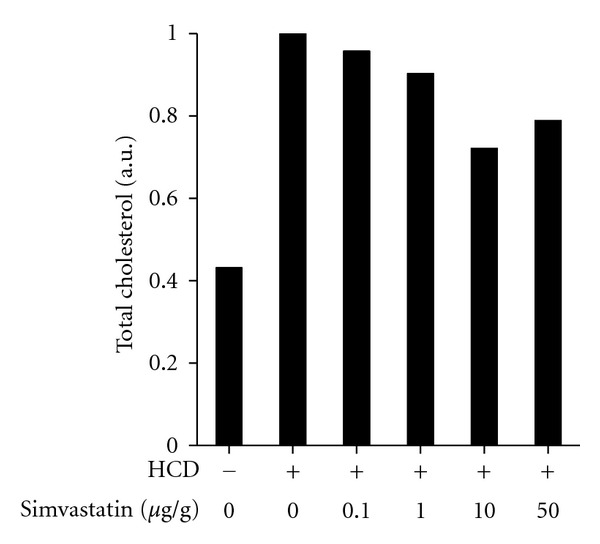
Effect of simvastatin on cholesterol levels in HCD-fed zebrafish larvae. Zebrafish larvae were fed a HCD with simvastatin mixed into zebrafish food at indicated quantities (*μ*g simvastatin per gram food) for 14 days. Total cholesterol was measured in lipid extracts from larvae homogenates. Values of total cholesterol per larva were normalized to the values in the group that received HCD only (second column). Homogenates of 20 animals were pooled together for each data point in each individual experiment. Experiments with 0, 10, and 50 *μ*g/g simvastatin were repeated twice, and average values from these two experiments are presented in the graph.

 To study the effect of simvastatin, we initially added it directly to water, as it has been reported in short-term studies [9]. However, we found that a prolonged exposure to simvastatin in water was toxic to zebrafish larvae. Varying the doses of simvastatin and the larval age at the beginning of treatment did not help improve zebrafish survival. Next, we mixed simvastatin into fish food at doses ranging from 0.1 to 50 *μ*g per gram of food (*μ*g/g). As estimated in Methods, a dose of, for example, 10 *μ*g/g simvastatin administered with zebrafish food is roughly an equivalent of a 50 mg simvastatin dose administered to human patients. As shown in Figure 2, these doses of simvastatin were less effective than expected from short-term studies, with no significant difference between the group that received HCD alone and the group that received HCD with higher doses of simvastatin (*P* = 0.11, *n* = 4 for the combined 10 and 50 *μ*g/g simvastatin group). As pharmacokinetics of simvastatin administered with food to zebrafish larvae has not been explored, these negative results could be due to a low effective dose of simvastatin. However, a higher dose is problematic given the toxicity of simvastatin added to water.

**Figure 3:**
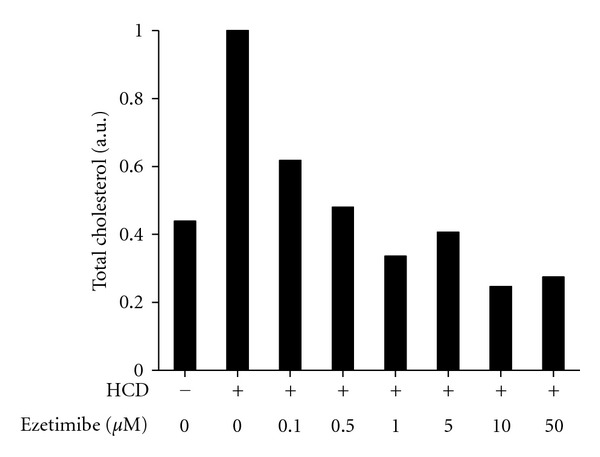
Effect of ezetimibe on cholesterol levels in HCD-fed zebrafish larvae. Zebrafish larvae were fed a HCD for 14 days. Ezetimibe was added at indicated concentrations directly to fish tank water, and the water was changed every day. Total cholesterol was measured in lipid extracts from larvae homogenates. Values of total cholesterol per larva were normalized to the values in the group that received HCD only (second column). Homogenates of 20 animals were pooled together for each data point in each individual experiment. Experiments with 0, 1, and 5 *μ*M ezetimibe were repeated three times, and average values from these three experiments are presented in the graph.

 In contrast to simvastatin, adding ezetimibe to water did not affect survival of larvae, their swimming pattern nor apparent food intake. Based on the range of doses of ezetimibe that has been reported to inhibit intestinal cholesterol absorption in zebrafish larvae [8], we tested 0.1–50 *μ*M ezetimibe added to water. We found that ezetimibe was very effective in reducing cholesterol levels in HCD-fed larvae (Figure 3). Already at the concentration of 1 *μ*M, ezetimibe reduced cholesterol to the levels observed in larvae fed control diet (*P* = 0.39 for the hypothesis that means of “control” and “HCD + ezetimibe” are different, *n* = 3).

 In humans, ezetimibe targets intestinal Niemann-Pick C1-like protein 1 (NPC1L1). The ezetimibe-binding domain in zebrafish Npc1l1 is highly homologues to the corresponding domain in human NPC1L1, with preserved phenylalanine and methionine required for high-affinity binding of ezetimibe [8]. Larvae treated with 6.25–50 *μ*M ezetimibe shows 25–75% reductions in gallbladder formation and in intestinal fluorescence derived from nitrobenzoxadiazole (NBD)-cholesterol [8]. These data suggest that the mechanism of inhibition of intestinal cholesterol absorption by ezetimibe in zebrafish is similar to that in humans, but direct evidence of the involvement of Npc1l1 in zebrafish cholesterol metabolism targeted by ezetimibe has not yet been reported.

**Figure 4:**
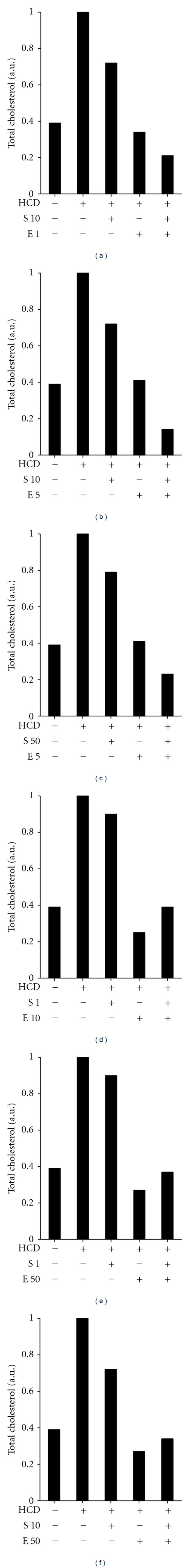
Effect of combined simvastatin and ezetimibe treatments on cholesterol levels in HCD-fed zebrafish larvae. Zebrafish larvae were fed a HCD for 14 days. Simvastatin and ezetimibe were administered as described in legends to Figures 1 and 2. Total cholesterol was measured in lipid extracts from larvae homogenates. Values of total cholesterol per larva were normalized to the values in the group that received HCD only (second column). Homogenates of 20–40 animals were pooled together for each data point in each individual experiment.

Next, we tested whether a combined treatment with ezetimibe (in water) and simvastatin (in food) would result in greater reductions in cholesterol levels in HCD-fed larvae. Concentrations of ezetimibe of 1 and 5 *μ*M, combined with 10 or 50 *μ*g/g simvastatin, reduced cholesterol to lower levels than individual treatments with ezetimibe or simvastatin (Figures 4(a), 4(b), and 4(c)), while combinations of higher concentrations of ezetimibe with simvastatin did not have any additive effect (Figures 4(d), 4(e), and 4(f)). Thus, even though simvastatin, under our experimental conditions, had little effect on cholesterol levels in zebrafish larvae, selected combinations of simvastatin with ezetimibe produced a greater effect in reducing cholesterol levels than each drug alone.

 Note, that in several experiments, treatments of HCD-fed zebrafish with ezetimibe or with a ezetimibe/simvastatin combination reduced cholesterol levels as much as 4-fold lower than in zebrafish that received control diet and no other treatment (Figures 3 and 4). Fish preferentially use lipids rather than carbohydrates as an energy source and would be classified, using standards applied to mammals, as hyperlipidemic and hypercholesterolemic, even when consuming their normal diet [10]. Treatments with ezetimibe and ezetimibe/simvastatin were able to reduce “normal” zebrafish hypercholesterolemia, without any apparent detrimental effects on larvae survival, swimming pattern, or feeding behavior.

## 4. Conclusions

 Cholesterol feeding of zebrafish larvae resulted in 2.5-fold increases in cholesterol levels in body fluids isolated from larvae homogenates. These results agree well with our earlier findings of elevated cholesterol levels in plasma of adult zebrafish fed a similar HCD and suggest that HCD-feeding likely induces hypercholesterolemia in larvae as well. We found that ezetimibe is well tolerated by zebrafish larvae and effectively reduces cholesterol levels in HCD-fed larvae to or even below the levels observed in control animals. In contrast, simvastatin added to water is poorly tolerated by zebrafish larvae and, when added to food at the doses tested, has little effect on cholesterol levels in HCD-fed larvae. Combination of low doses of ezetimibe and simvastatin may have an additive effect in reducing cholesterol levels in zebrafish. These results suggest that, similar to its therapeutic effect in humans, ezetimibe effectively reduces cholesterol levels in HCD fed zebrafish larvae and can be used in the hypercholesterolemic zebrafish model to study its effects on vascular lipid accumulation and inflammation, processes relevant to the pathogenesis of human atherosclerosis.
